# The anti-inflammatory effects of photobiomodulation are mediated by cytokines: Evidence from a mouse model of inflammation

**DOI:** 10.3389/fnins.2023.1150156

**Published:** 2023-04-06

**Authors:** Shirin Shamloo, Erwin Defensor, Peter Ciari, Gaku Ogawa, Laura Vidano, Jennifer S. Lin, John A. Fortkort, Mehrdad Shamloo, Annelise E. Barron

**Affiliations:** ^1^Department of Bioengineering, Schools of Medicine and Engineering, Stanford University, Stanford, CA, United States; ^2^Department of Neurosurgery, School of Medicine, Stanford University, Stanford, CA, United States

**Keywords:** photobiomodulation (PBM), lipopolysaccharide (LPS), light therapy, neuroinflammation, systemic inflammation, neurodegenerative disorders

## Abstract

There is an urgent need for therapeutic approaches that can prevent or limit neuroinflammatory processes and prevent neuronal degeneration. Photobiomodulation (PBM), the therapeutic use of specific wavelengths of light, is a safe approach shown to have anti-inflammatory effects. The current study was aimed at evaluating the effects of PBM on LPS-induced peripheral and central inflammation in mice to assess its potential as an anti-inflammatory treatment. Daily, 30-min treatment of mice with red/NIR light (RL) or RL with a 40 Hz gamma frequency flicker for 10 days prior to LPS challenge showed anti-inflammatory effects in the brain and systemically. PBM downregulated LPS induction of key proinflammatory cytokines associated with inflammasome activation, IL-1β and IL-18, and upregulated the anti-inflammatory cytokine, IL-10. RL provided robust anti-inflammatory effects, and the addition of gamma flicker potentiated these effects. Overall, these results demonstrate the potential of PBM as an anti-inflammatory treatment that acts through cytokine expression modulation.

## Introduction

Photobiomodulation (PBM) is a technique that uses low-intensity light for therapeutic purposes. Numerous salutary effects have been ascribed to PBM, including reduced oxidative stress (Dos Santos et al., 2017; Heo et al., 2019), improved cerebral blood flow (Salgado et al., 2015; Hipskind et al., 2019; Baik et al., 2021), and widespread anti-inflammatory effects *via* reduction of inflammatory cytokines such as IL-8, IL-6, and IL-1β (Hamblin, 2017; Xie et al., 2022). PBM promotes neurogenesis in murine models of traumatic brain injury (TBI) and stroke (Xuan et al., 2014; Cassano et al., 2016; Yang et al., 2018) while also attenuating motor deficits in TBI mouse models. Various parameters of PBM are configurable, including wavelength and pulse mode (Zein et al., 2018). The use of pulsed or flickered light can impact the effectiveness of a PBM treatment and has been applied to produce additional therapeutic benefits (Iaccarino et al., 2016). For example, the application of PBM with 40 Hz gamma frequency oscillations decreased amyloid-β (Aβ) loads and increased the recruitment and activation of microglia in mouse models of Alzheimer’s disease (Singer et al., 2018; Adaikkan et al., 2019; Martorell et al., 2019). Additionally, PBM at specific frequencies (30, 40, or 50 Hz) displayed neuroprotective effects in a two-vessel occlusion (2VO), an animal model of brain ischemia by preventing neuronal degeneration in the CA1 region of the hippocampus, while also improving spatial learning and reference memory following the ischemic episode (Zheng et al., 2020). These results suggested that slow gamma light may lessen neuronal injury after global brain ischemia.

Additionally, NF-κB and mitogen-activated protein kinase (MAPK) pathways are critical mediators of inflammatory responses and regulate cytokine expression and release (Cloutier et al., 2007; de Souza et al., 2009; Bachstetter and Van Eldik, 2010; Lawrence and Fong, 2010; Hayden and Ghosh, 2014; Liu et al., 2018; Xiao et al., 2020). It has been shown that PBM with 40 Hz flicker leads to phosphorylation of NF-κB and MAPK in mice and increases the expression of some cytokines compared to no-light groups with induced inflammation (Garza et al., 2020). Singer et al. investigated the effects of exposing mice to PBM for durations of 5 min to 1 h, comparing the effects of constant (non-flickered) white light with light flickered at either 20 or 40 Hz or with a random flicker (Singer et al., 2018), using LPS-treated mice with no exposure to PBM as a comparison. They reported that exposure to PBM with gamma flicker induced changes in certain pro- and anti-inflammatory cytokines in the brain. Specifically, the proinflammatory cytokines IL-6, IFN-γ, and IL-1β were upregulated in the 40 Hz light flicker groups when compared to the 20 Hz and constant light groups. Overall, the findings of Singer et al. suggested that PBM and PBM with 40 Hz light flicker can alter cytokine expression and neuroinflammatory processes beneficially and in a pattern distinct from acute neuroinflammation.

Systemic inflammation accompanies infection and major surgical procedures and is a significant risk factor for dementia, acute delirium, and neurodegenerative diseases. This risk increases with age (Martins and Fernandes, 2012). Several *in vivo* and *ex vivo* models have been established to investigate the pathophysiology of neuroinflammation-mediated brain injury and to evaluate the efficacy of therapeutic strategies for treatment (Kiers et al., 2017; Seemann et al., 2017). Administration of LPS has been used to induce neuroinflammation to model neuroinflammatory-mediated neurodegeneration (Batista et al., 2019). LPS, a cell wall component of gram-negative bacteria (Rietschel et al., 1982), stimulates microglia proliferation *via* NF*-*κB activation in mice, leading to cognitive impairments in learning and memory tasks, as well as impaired motor function in pole tests. Furthermore, it has been reported that direct LPS brain exposure causes neuronal degeneration in specific brain nuclei. Thus, murine models of LPS-induced systemic inflammation and neuroinflammation are relevant tools for investigating neuroinflammation associated with neurodegenerative diseases and for testing potential anti-inflammatory therapeutics. This experimental model mimics some aspects of the underlying inflammation seen in neurodegenerative disorders (Howcroft et al., 2013). While anti-inflammatory and immunomodulatory properties of PBM in improving cognition have been demonstrated in some particular contexts (Ferraresi et al., 2015; Cassano et al., 2016; Dos Santos et al., 2017; Hamblin, 2017; Yang et al., 2018; Heo et al., 2019; Hipskind et al., 2019; Heinig et al., 2020), the pathways underlying the immunomodulatory effects of PBM have yet to be elucidated clearly in an *in vivo* murine study.

The aim of the current study was to employ a well-established murine LPS model to evaluate the effects of PBM, predominantly red/NIR light (640 and 880 nm), with and without 40 Hz gamma flicker, on systemic and central inflammation and expression of 48 different inflammatory cytokines. Through the induction of inflammation *via* LPS, we aimed to discern the mechanistic pathways responsible for the immunomodulatory properties of PBM and to determine its potential efficacy as an experimental therapeutic and prophylactic tool for the treatment of neurodegenerative disorders.

## Results

### Red/NIR light or red/NIR light with 40 Hz flicker did not alter the appearance and general behavior of mice

**Figure 1:**
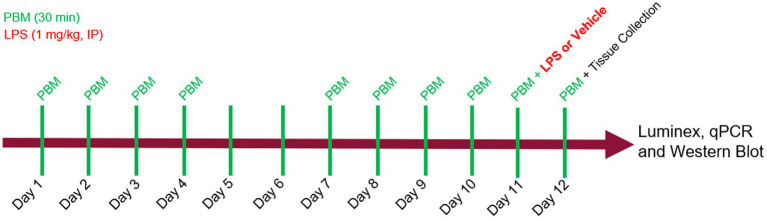
Experimental design. Photobiomodulation (PBM) was administered daily, for 30 min per day, on days 1 through 5 and days 8 through 12. On day 11, mice received either LPS (1 mg/kg) or vehicle injection, IP, 30 min after PBM. On day 12, mice received a final PBM treatment before tissue was collected at 24 hrs after LPS. Luminex, qPCR and western blot assays were performed on brain and plasma samples.

**Figure 2:**
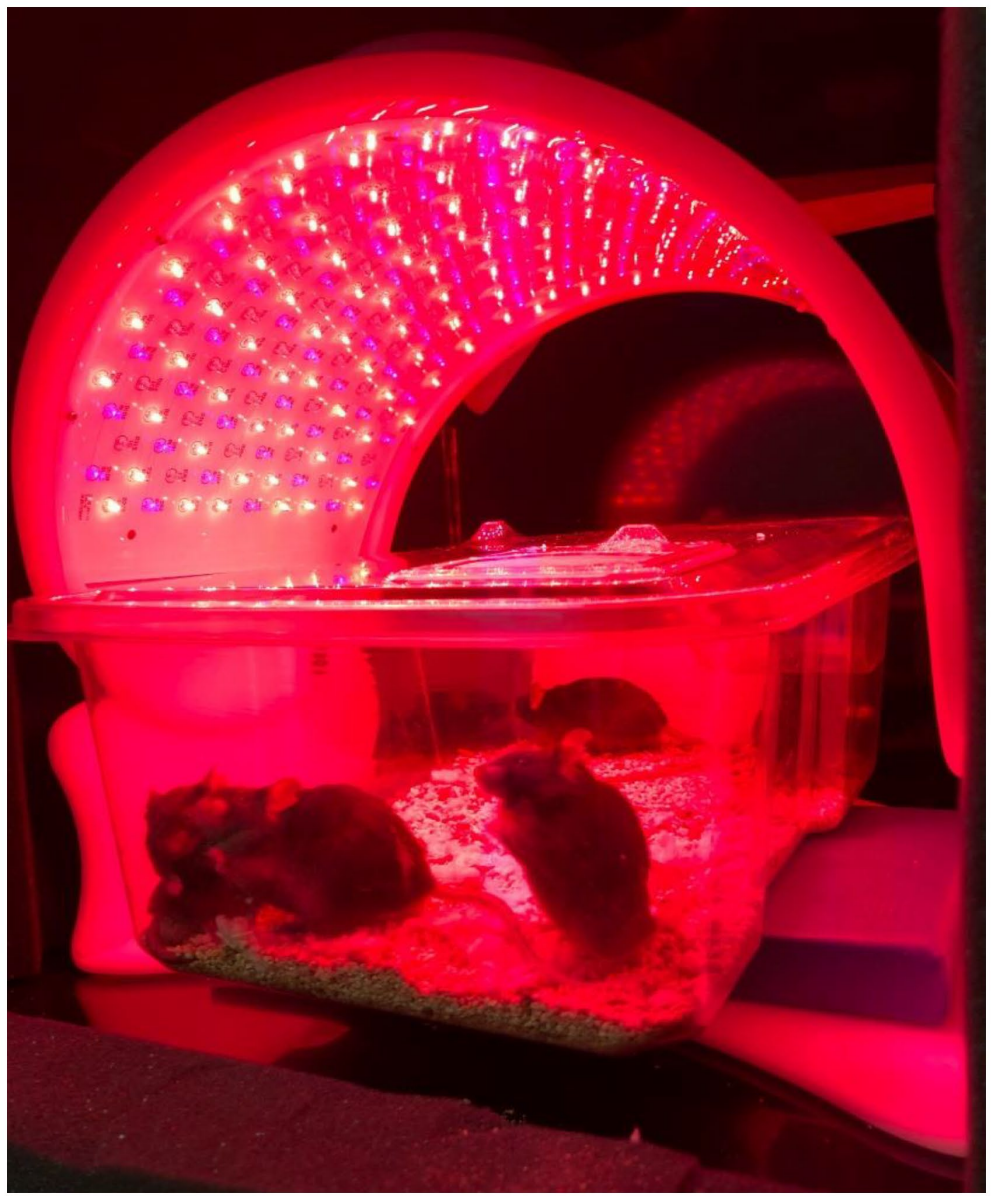
Apparatus setup. Auragen light therapy units contain a matrix of LEDs, which consists of an array of three different types of LED bulbs that can emit light either at 640 nm (red), 880 nm (NIR), and 465 nm (blue turquoise), respectively.

PBM was administered in 30-min sessions to the mice over the course of 12 days, for 5 consecutive days each week (Figure 1). Refer to Materials and methods for details on study design. Treatment times were selected based on efficacy observed in pilot studies and adapted from previous studies (Garza et al., 2020). Mice received either no PBM (NL), PBM with red/NIR light (RL), or PBM with red/NIR light and 40 Hz gamma flicker (RLG). On day 11 of the experiment, mice were dosed IP with either vehicle (saline) or LPS (1 mg/kg) following the daily PBM treatment. On day 12, the final PBM treatment session was performed and was followed by tissue collection at 24 h post LPS/vehicle injection. Auragen® light therapy units (Reversal Solutions, Inc.), which consist of a matrix of LEDs, were used for the PBM treatment by placing the transparent mouse cage at the base of the device and then isolating each device from other units by using custom built isolation chambers (Figure 2; Supplementary Figure S1). Mice were briefly observed in the home cage on each treatment day to detect any gross behavioral or physical effects of the PBM therapy and to confirm the health of the mice. All mice survived the duration of the experiment, with no gross behavioral or physical abnormalities detected.

### Red/NIR light and red/NIR light with 40 Hz flicker differentially alter cytokine protein expression profiles in the brain and plasma

**Figure 3:**
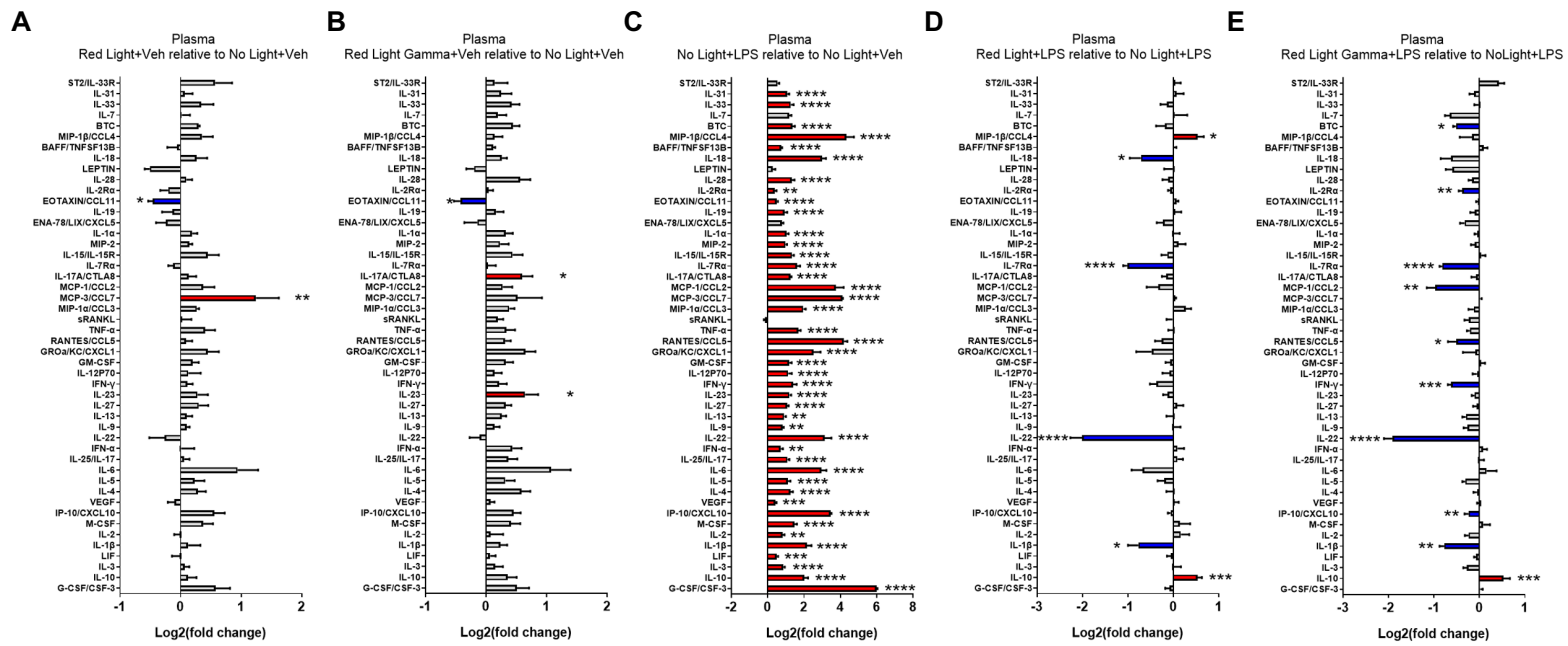
Log2 fold change of plasma cytokines measured in the Luminex assay depicts the effects of **(A)** red light, **(B)** red light gamma, and **(C)** LPS relative to no light + vehicle, as well as effects of **(D)** red light and **(E)** red light gamma relative to no light + LPS. Data are presented as mean ± SEM. ^*^p < 0.05, ^**^p < 0.01, ^***^p < 0.001, ^****^p < 0.0001.

**Figure 4:**
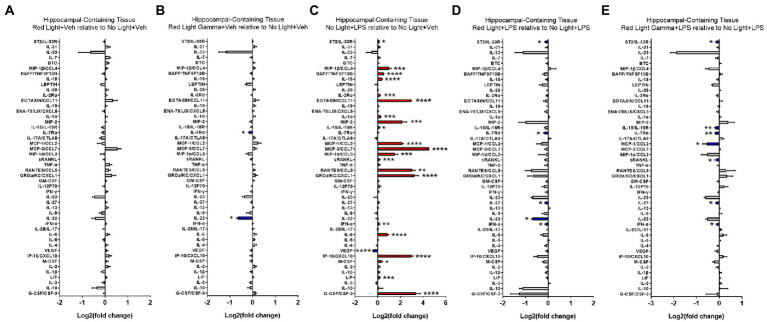
Log2 fold change of cytokines in hippocampal-containing brain tissue measured in the Luminex assay depicts the effects of **(A)** red light, **(B)** red light gamma, and **(C)** LPS relative to no light + vehicle, as well as effects of **(D)** red light and **(E)** red light gamma relative to no light + LPS. Data are presented as mean ± SEM. ^*^p < 0.05, ^**^p < 0.01, ^***^p < 0.001, ^****^p < 0.0001.

To evaluate the effects of RL and RLG on cytokine protein expression, groups receiving these PBM treatments with vehicle were compared to the NL vehicle group. The cytokine changes induced by RL in the plasma are presented in Figure 3A and select cytokines in Supplementary Figure S2, while changes in the brain are presented in Figure 4A and select cytokines in Supplementary Figure S3. RL produced mild changes in cytokine expression, with statistically significant differences only in plasma and not the brain, for EOTAXIN/CCL11 (*p* < 0.05) and MCP-3/CCL7 (*p* < 0.01). The cytokine changes induced by RLG in the plasma are presented in Figure 3B and Supplementary Figure S2. The changes in the brain are shown in Figure 4B and Supplementary Figure S3. In the plasma, RLG produced an altered pattern of cytokine expression, which included statistically significant changes in IL-23 (*p* < 0.05), IL-17A (*p* < 0.05), and EOTAXIN/CCL11 (*p* < 0.05). In the brain, the cytokine response to RLG was limited, with significant decreases in IL-7Rα (*p* < 0.05) and IL-22 (*p* < 0.05).

### LPS induces a proinflammatory cytokine protein expression in brain and plasma

To evaluate the effect of LPS on cytokine protein expression, NL with LPS treatment was compared to NL with vehicle treatment. The cytokine changes induced by LPS in the plasma are presented in Figure 3C and Supplementary Figure S2, while changes in the brain are shown in Figure 4C and Supplementary Figure S3. As expected, LPS induced robust cytokine responses in both the plasma and brain, with greater overall changes observed in the plasma. The cytokine profile induced by LPS in the plasma is consistent with a systemic inflammatory response with statistically significant increases in 43 of the 48 cytokines measured (Figure 3; Supplementary Figure S2). In the brain, statistically significant increases were detected in 21 of the 48 cytokines measured (Figure 4; Supplementary Figure S3).

### Red light and red light with 40 Hz flicker modulate LPS-induced cytokine protein expression

To evaluate the effects of RL and RLG on LPS-induced cytokine protein expression, groups that received these PBM treatments and were then challenged with LPS were each compared to the NL group that was challenged with LPS. The modulatory effects of RL on LPS-induced cytokine expression in the plasma are presented in Figure 3D, Supplementary Figure S2 while changes in the brain are presented in Figure 4D and Supplementary Figure S3. In the plasma, RL modulated LPS induction of MIP-1β (*p* < 0.05), IL-10 (*p* < 0.001), IL-18 (*p* < 0.05), IL-7Rα (*p* < 0.0001), IL-22 (*p* < 0.0001), and IL-1β (*p* < 0.05). In the brain, RL modulated LPS induction of ST2 (*p* < 0.05), IL-7Rα (*p* < 0.05), IL-27 (*p* < 0.05), IL-22 (*p* < 0.05), and IFN-α (*p* < 0.05).

**Figure 5:**
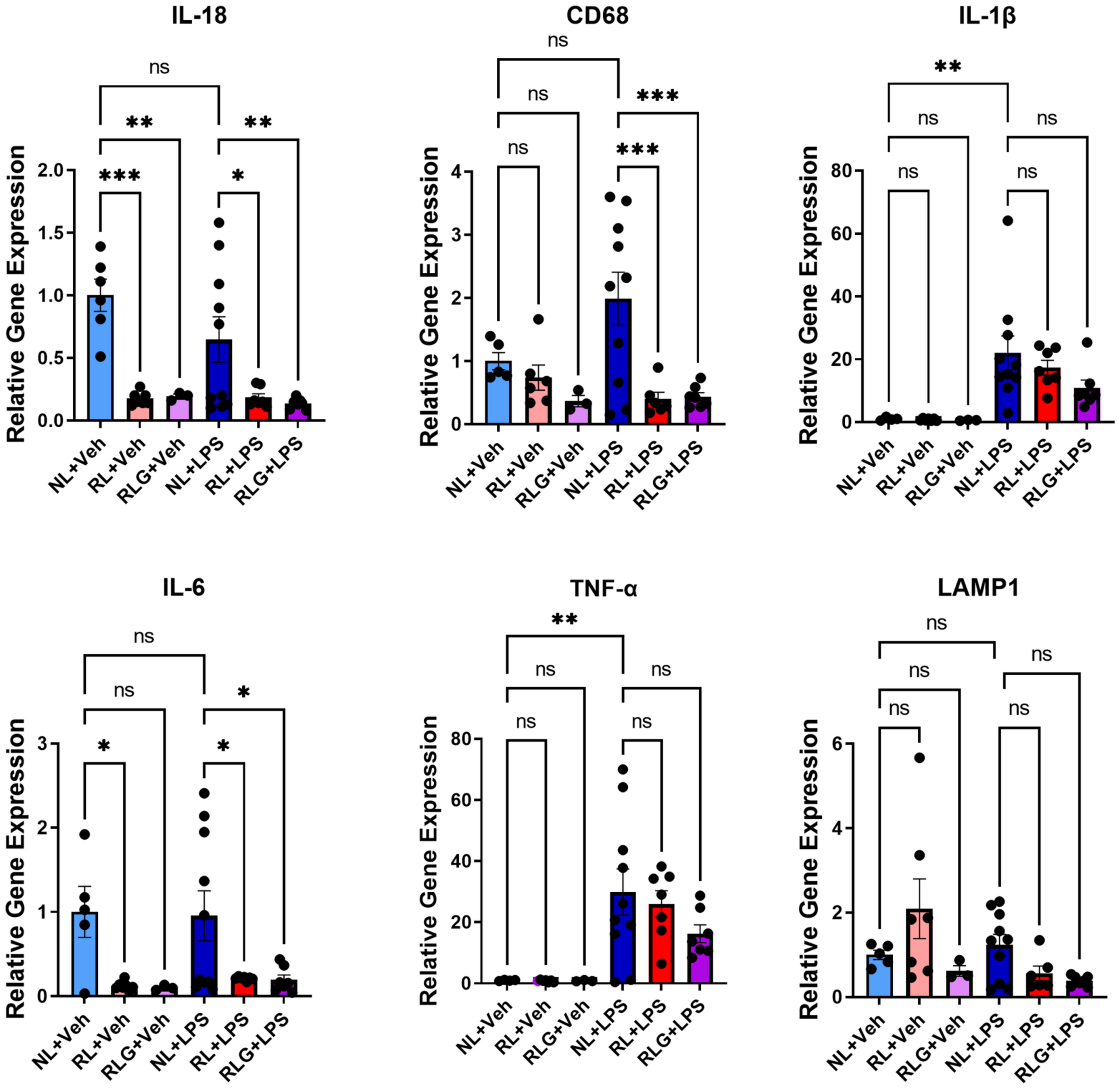
RT-qPCR analysis of select markers in hippocampus-containing brain tissue. There was significant downregulation of IL-18, CD68, and IL-6 in the RL + LPS and RLG + LPS groups, when compared to the NL + LPS group. In addition, when compared with the NL + Veh group, there was a significant downregulation of IL-18 in both the RL + Veh and the RLG + Veh groups, as well as a downregulation of IL-6 in the RL + Veh group. Upregulation of TNF-α and IL-1β was consistent with an inflammatory response in the NL + LPS group, when compared to the NL + Veh group. Data are presented as mean ± SEM. ^*^p < 0.05, ^**^p < 0.01, ^***^p < 0.001, ^****^p < 0.0001.

The modulatory effects of RLG on LPS-induced cytokine protein expression in the plasma are presented in Figure 3E and Supplementary Figure S2. The changes in the brain are shown in Figure 4E and Supplementary Figure S3. In the plasma, RLG modulated LPS induction of BTC (*p* < 0.05), IL-2Rα (*p* < 0.01), IL-7Rα (*p* < 0.0001), MCP-1 (*p* < 0.01), RANTES (*p* < 0.05), IFN-γ (*p* < 0.001), IL-22 (*p* < 0.0001), IP-10 (*p* < 0.01), IL-1β (*p* < 0.01), and IL-10 (*p* < 0.05). In the brain, RLG modulated LPS induction of ST2 (p < 0.05), IL-15 (*p* < 0.01), IL7-Rα (*p* < 0.01), MCP-1 (*p* < 0.05), sRANKL (*p* < 0.05), IL-27 (*p* < 0.05), and IFN-α (*p* < 0.05).

### Red light or red light with 40 Hz flicker did not alter AKT, ERK, or GSK3β

In addition to the cytokine analyses by Luminex assay, protein levels of AKT, phospho-AKT, GSK-3β, phospho-GSK3β, ERK 1/2, phospho-ERK 1/2, ATG5, phospho p70S6k, and p70S6k were evaluated by western blot (WB). No significant differences between the treatment groups were detected for the proteins measured by WB (Supplementary Figures S4–S7).

### Red light and red light with 40 Hz flicker modulate cytokine gene expression

Based on the protein expression observations, we next investigated gene expression changes in hippocampal-containing brain tissue by RT-qPCR, for a select subset of cytokines, as well as CD68 and LAMP1 (Figure 5). As previously noted, RL did not induce significant changes in cytokines at the protein level in brain tissue. In contrast, at the mRNA level, RL downregulated IL-18 (*p* < 0.0001) and IL-6 (*p* < 0.05), while RLG also downregulated IL-18 (*p* < 0.01) and showed a similar, but not statistically significant, trend to decrease IL-6. In mice challenged with LPS, RL and RLG pretreatment both also downregulated IL-18 (*p* < 0.05 and < 0.01, respectively), IL-6 (*p* < 0.05 for both) and CD68 (*p* < 0.001 for both).

## Discussion

Systemic LPS exposure induces widespread systemic and central inflammation and induction of inflammatory cytokines (Meneses et al., 2018). Here we report an investigation of the therapeutic potential of PBM, specifically 10 days of red/NIR light exposure at 640 and 880 nm for 30 min per day, either with steady light or with gamma-flickered light, following systemic LPS exposure in mice and the impact of PBM on systemic and central inflammation and expression of 48 different inflammatory cytokines (as measured by Luminex array analysis). We found that pretreatment with PBM using red/NIR light has both systemic and central anti-inflammatory properties and that PBM acts through cytokine expression modulation as its mechanism. PBM downregulated LPS induction of key proinflammatory cytokines, such as IL-1β and IL-18, and upregulated the key anti-inflammatory cytokine IL-10. The current study shows that regular exposure to PBM for 30 min, 5 days per week, over 2 weeks has therapeutic immunomodulatory effects in mice following systemic LPS exposure.

Systemic administration of LPS produced a robust inflammatory response, indicated by upregulation of the majority of cytokines evaluated in the panel, and pretreatment with either RL or RLG partially attenuated the LPS-induced cytokine expression. PBM led to the downregulation of inflammatory cytokines responsible for the recruitment of immune cells to the sites of inflammation and injury. We saw a downregulation of IL-22, an epithelial proinflammatory cytokine. Furthermore, the light treatment led to the downregulation of IFN-γ, a key modulator of macrophage activity and proliferation and a major switch for the activation of the innate immune response. Additionally, PBM led to the downregulation of IL-7Rα, the receptor for the inflammatory cytokine IL-17A, which is secreted by T-lymphocytes. We observed the downregulation of CCL2, a cytokine responsible for the recruitment of immune cells to sites of inflammation, and CCL5, another chemoattractant that promotes the inflammatory response. BTC, a gene that encodes the expression of several epidermal growth factor proteins, was also downregulated by PBM. Downregulation of IL-2Rα, proteins that regulate the functionality of T cells, was also observed.

It is known that LPS exposure leads to activation of the inflammasome pathway, as evidenced by upregulation of IL-1β and IL-18 (Ganz et al., 2011). LPS is a ligand for TLR4, which induces a downstream signaling cascade and inflammasome activation *via* activation of NF-κB and the subsequent release of IL-1β and IL-18. Specifically, the NLRP3 protein can be activated by extracellular signals, such as LPS, ultimately cleaving pro-IL-1β into bioactive IL-1β (Ren and Torres, 2009). Here, we report that exposure to PBM may lead to downregulation of inflammasome activation. We observed downregulation of IL-1β, a proinflammatory cytokine, the secretion of which depends upon inflammasome activity. The downregulation of IL-1β seen in the RL/RLG groups suggests that PBM has the capacity to potentially alter the inflammasome pathway. IL-18, the critical proinflammatory cytokine that promotes T cell activation and induces other cytokines such as IFN-γ, was also downregulated. The downregulation of IL-18, especially in conjunction with the downregulation of IL-1β, strongly indicates that sustained light exposure has some impact on the reduction of inflammasome activation triggered by LPS exposure. PBM also upregulated IL-10 in the RL and RLG groups following LPS challenge. The cytokine IL-10 has anti-inflammatory and immunosuppressive properties that reduce tissue damage as the host organism mounts an immune response (Ling et al., 2011). Our results thus demonstrate that PBM induces certain systemic anti-inflammatory responses by upregulating protective cytokines such as IL-10.

As previously mentioned, it has been shown that PBM with 40 Hz flicker leads to phosphorylation of NF-κB and MAPK in mice and produces increases in the expression of some cytokines compared to NL LPS groups (Garza et al., 2020). In the study by Singer et al., they found upregulation of proinflammatory cytokines IL-6, IFN-γ and IL-1β in their 40 Hz light flicker groups when compared to the 20 Hz groups, while random flicker groups saw the highest upregulation of IL-10 (Singer et al., 2018). Both our experimental design and results differ from those of the Singer research group, although we also saw modulation of certain cytokines with exposure to RL only (specifically in CCL11 and in CCL7). We also saw increases in cytokine expression of IL-23 and IL-17A and downregulation of CCL11, IL-17Rα, and IL-22 in our RLG groups. These differences in cytokine expression mediated by light may be due to our use of predominantly red and NIR light, our increased treatment time either in the individual sessions (15 vs. 30 min), the overall duration of the study (1 vs. 10 days), or differences in mode of exposure to PBM. Their PBM treatment devices were laboratory-made, whereas the light units that we used are standardized as the Auragen system is a commercial product.

Here we also found that exposure of mice to PBM prior to LPS administration led to further upregulation of CCL4. CCL4 is a proinflammatory cytokine with chemokine activity leading to the recruitment of NK cells and monocytes to inflammation and injury sites. This suggests that PBM using red light can trigger innate immune cell responses at the systemic level. A similar observation was reported by Singer et al. following a single exposure to 40 Hz white-light stimulation of male mice (Singer et al., 2018). However, it is possible that an initial increase in the innate immune response, as indicated by an increase in the levels of IFN-γ, would be reflected in the shorter treatment intervals explored by the Singer group. By contrast, we may be seeing that our study’s comparatively longer-term exposure to predominantly red/NIR light (640 and 880 nm), with or without a 40-Hz flicker, can ultimately result in an overall decrease in systemic inflammation, even while stimulating white blood cell responsiveness. It is also important to note that the Singer group examined visual cortex tissue samples only, while we investigated hippocampus-containing brain tissue as well as peripheral blood. Potential differences in local (visual cortex) vs. systemic and central inflammatory responses should thus be considered. Also, Singer et al. used white light bulbs, while our study used predominantly red and NIR LED light sources with precise wavelengths (640 and 880 nm), which may also account for different physiological effects (Singer et al., 2018). Consistent with all aforementioned peripheral changes, we observed central upregulation of important CNS cytokines IFN-α, IL-15, sRANKL, and IL-33R within 24 h of LPS exposure. We found that pre-exposure to 30 min of red/NIR light daily for 10 consecutive days can prevent or reduce this induction significantly. Furthermore, the PBM-mediated reduction in neuroinflammation induced by peripherally administered LPS in the current study is consistent with a previous study reporting that PBM protected against dopaminergic cell death and associated microglia-evoked neuroinflammation induced by intracranially administered LPS in rats (O'brien and Austin, 2019).

Our results are also consistent with *in vitro* studies on human cell lines investigating the anti-inflammatory effects of PBM. Aguida et al. found that daily exposure of HEK293 cells to two 10-min, high-intensity periods of IR light caused a significant reduction in the TLR-4 dependent inflammatory response pathway (Aguida et al., 2021). In particular, within 2 days of treatment, they found that exposure to the light source resulted in a significant decline in NF-κB and AP1 activity, and decreased expression of IL-6, IL-8, TNF-α, IFN-α, and IFN-β. They hypothesized that PBM affects underlying cellular mechanisms involving modulation of ROS, thus downregulating the inflammatory immune response. Similarly, Nejatifard et al. reviewed the effects of the coronavirus on alveolar epithelial cells and the mitigation of those effects by PBM (Nejatifard et al., 2021). The cytokine storm induced by the coronavirus activated macrophages and neutrophils, causing an upregulation of inflammatory cytokines and markers such as TNF-α, IL-1β, IL-6, ICAM-1, MIP-2, iNOS, and ROS, and a downregulation of the anti-inflammatory cytokine IL-10. In severe cases, this resulted in multi-organ failure and acute respiratory distress syndrome (ARDS). The use of PBM was found to downregulate TNF-α, IL-1β, IL-6, ICAM-1, MIP-2, iNOS, and ROS, and upregulate IL-10, thus reducing inflammation, improving oxygenation and promoting tissue regeneration. In our study, although using an *in vivo* animal model instead of human cell lines, we also observed downregulation of IL-6 gene expression (although not TNF-α) and upregulation of IL-10 while the LPS-induced increases in both IL-1β and IFN-α were mitigated by light treatment.

Much is already known about the underlying mechanisms of PBM. PBM is currently believed to exert its physiological effects on complex biological systems primarily through the photon-induced release of nitric oxide (NO) from cytochrome C oxidase (Cox) chromophores within mitochondria (also known as complex IV within the mitochondrial electron transport chain; Cardoso et al., 2022; Oh et al., 2022; Pope and Denton, 2022). This enzyme, which contains both heme and copper centers, absorbs light in the red and NIR regions of the spectrum (Karu, 2010). Photons dissociate inhibitory NO from the enzyme, leading to an increase in electron transport, mitochondrial membrane potential, and ATP production (Hamblin, 2018; Dompe et al., 2020). The release of NO can also generate reactive oxygen species as well as various anti-inflammatory molecules. Another proposed mechanism of action suggests that PBM activates light-sensitive ion channels, thereby allowing calcium to enter cells. After initial photon absorption, various signaling pathways are activated *via* reactive oxygen species (ROS), cyclic AMP, NO, and Ca^2+^, thus activating a variety of transcription factors. These transcription factors, in turn, result in the increased expression of genes related to anti-inflammatory signaling, protein synthesis, cell migration, and proliferation, anti-apoptotic proteins, antioxidant enzymes (de Freitas and Hamblin, 2016; Dompe et al., 2020). Previous data also shows that PBM directed at the abdomen can alter the gut microbiome in mice, providing beneficial immunomodulation (Chen et al., 2021). The importance of the gut microbiome in diseases associated with neuroinflammation highlights the importance of characterizing broader potential mechanisms of action, such as in determining whether light is absorbed by microbial or gut epithelial cells or whether changes in the microbiome are due to anti-inflammatory or redox signaling changes induced by PBM (Liebert et al., 2019). In this initial study, our goal was to examine the potential systemic and anti-inflammatory effects of PBM treatment against an inflammatory challenge—intraperitoneal LPS injection—focusing on treatment with red/NIR light, with or without a 40 Hz gamma flicker.

We acknowledge the limitations of our study. At least at the systemic level, apparent anti-inflammatory effects were also accompanied by an apparent uptick in the activation of innate immune cells such as macrophages and NK cells. It is possible that PBM affects only specific pathways in the LPS-induced inflammatory cascade, such as the inflammasome, while other forms of inflammation, such as those seen in the progressive and more advanced stages of neurodegenerative diseases, might not be affected. It is also possible that certain parameters of PBM, such as the exposure time or specific wavelengths used, could determine the precise modulation of anti-inflammatory and innate immune cell activation pathways. It may also be the case that PBM, especially in the presence of gamma flicker, may itself provoke a certain level of inflammation (such as activation of microglia), while also serving as a systemic anti-inflammatory treatment that protects against LPS-induced damage (which can be seen as a surrogate for the effects of a bacterial infection, for instance). In some cases, we observed changes at the mRNA level that we were not able to confirm at the protein level; this implies that it is possible that the time interval between the administration of the LPS and tissue collection was not long enough for protein synthesis to be thoroughly carried out. In future studies, it may be helpful to implement a more extended period between the initiation of neuroinflammation and tissue collection. This may also be part of the reason why we saw local changes in cytokine levels, but not at the systemic level. In further studies, it may also be useful to have multiple groups with varying lengths of treatment time to determine whether the “dose” of light treatment affects cytokine levels differently (i.e., locally vs. systemically). Further, the flickering light treatment (RLG) using a 40-Hz gamma flicker is equivalent, temporally, to approximately a half-dose of the light provided by steady-state RL treatment, and this may have influenced the results we saw between RL and RLG groups. Singer et al. accounted for this discrepancy by including a random flicker light group. This is something to account for in future studies, either by adjusting the length of treatments accordingly or by altering the light units themselves, which cannot presently provide a random flicker. Additionally, we looked only at male mice; this serves as a limitation given the effect of sex on the immune response.

Here we report that 10 days of red/NIR light exposure at 640 and 880 nm for 30 min per day, either with steady light or with gamma-flickered light, has potential anti-inflammatory properties in naïve mice, as well as following LPS challenge both in the brain and systemically. We found that PBM modulates cytokine expression for its mechanism of action and may downregulate the inflammasome activation pathway. Future studies will be needed to explore the time course and temporal profile of the impact of light on inflammatory cytokines and response following LPS exposure. Additionally, further research is required to understand the response following other pathological neuroinflammatory stimuli besides LPS, such as injury-induced and/or misfolded protein response in neurodegeneration models. Overall, the results of our study provide a promising indication of the anti-inflammatory immunomodulatory potential of PBM in treating neurological disorders in which inflammation is a factor in disease progression.

## Materials and methods

### Animals

Male C57BL/6 J mice were obtained from Jackson Laboratories (Stock Number 000664). Mice were between 11 and 13 weeks old during the experiment and were group-housed in cages with 4 or 5 mice total, under a reverse light cycle with lights off at 8:30 a.m. and lights on at 8:30 p.m. Mice were allowed to acclimate for 1 week upon arrival. In the week prior to the experiment, all mice (including those in the control group) were handled daily, each individually for 5 min, in the room where light therapy treatments would be conducted, for 5 days total. On the days PBM was administered, mice were moved to a holding area adjacent to the separate treatment room. The treatment room and holding area were illuminated with red light. Food and water were always freely available to the mice, except for the 30 min while they were in the light therapy chambers. All procedures related to animal maintenance and experimentation were approved by the Stanford University Administrative Panel for Laboratory Animal Care and conformed to the U.S. National Institutes of Health Guide for the Care and Use of Laboratory Animals. Efforts were made to minimize the number of mice used and their suffering. The total number of mice per group for each of the control and treatment groups is given in Table 1.

**Table 1:** Details of treatment groups and group names.

Treatment	Group name abbreviation	Treatment duration	Housing	# of male mice
No light + vehicle (0.9% saline)	NL vehicle	N/A	Group housed (5/cage)	*n* = 10
No light + LPS (1 mg/kg)	NL LPS	N/A	Group housed (4 or 5/cage)	*n* = 14
Red/NIR light only + vehicle (0.9% saline)	RL vehicle	2 weeks (30 min × 5 days per week)	Group housed (4 or 5/cage)	*n* = 14
Red/NIR light only + LPS (1 mg/kg)	RL LPS	2 weeks (30 min × 5 days per week)	Group housed (4 or 5/cage)	*n* = 14
Red/NIR light w/gamma flicker + vehicle (0.9% Saline)	RLG vehicle	2 weeks (30 min × 5 days per week)	Group housed (4 or 5/cage)	*n* = 14
Red/NIR light w/gamma flicker + LPS (1 mg/kg)	RL LPS	2 weeks (30 min × 5 days per week)	Group housed (4 or 5/cage)	*n* = 14

### Experimental overview

PBM was administered in 30-min sessions to the mice over the course of 12 days, for 5 consecutive days each week (Monday through Friday, with no treatment on days 6 and 7; Figure 1). Treatment times were selected based on efficacy observed in pilot studies and adapted from previous studies (Garza et al., 2020). Mice were group-housed (*n* = 4 or 5 per cage) and received either no PBM (NL), PBM with red/NIR light (RL), or PBM with red/NIR light and 40 Hz gamma flicker (RLG). On day 11 of the experiment, mice were dosed IP with either vehicle (saline) or LPS (1 mg/kg, Sigma-Aldrich, Cat. No: 93572–42-0) following the daily PBM treatment. On day 12, the final PBM treatment session was performed and was followed by tissue collection at 24 h post LPS/vehicle injection.

### Photobiomodulation treatment

The Auragen® light therapy units were provided by Reversal Solutions, Inc. as a gift. Transparent mouse cages (Innovive) were placed at the base of the apparatus, which is connected to a curved LED panel that extends above the base (Figure 2). The LED panel consists of an array of 328 individual LEDs, including 86 NIR (880 nm) LEDs, 108 blue turquoise (465 nm) LEDs, and 134 red (640 nm) LEDs (additional details on the LED technical specifications can be found in Supplementary material). The LEDs are arranged in alternating rows of gradually increasing length (from 9 to 12 units), moving from either end of the LED panel toward its center. Each Auragen light therapy unit is equipped with a controller that allows the user to select between different predefined programs of use. These programs include three different wavelength modes (“Renew,” “Calm,” and “Relief”) and three different flicker modes (theta flicker, gamma flicker, and no flicker), thus providing a total of nine different modes of use. Each mode has a programmed duration of 30 min. In the current study, the “Renew” setting, which utilizes predominantly red and NIR LEDs, was used either with gamma flicker (which we termed RLG treatment) or without gamma flicker (RL treatment). Additional details and descriptions of Auragen light therapy units are available from the manufacturer. The units offer the capacity to apply pulsed sound, including gamma- or theta-flickered sound that synchronizes with the light flicker, but we applied only light, not sound, in the present study. Six light therapy units were installed into separate custom isolation chambers fabricated from acrylonitrile butadiene styrene (ABS) plastic (Supplementary Figure S1), which served to segregate light and light flicker from the surrounding units. Light units were used in their normal programmed modes. RL and RLG treatments were each administered for 30 min, as programmed.

### Tissue collection

For terminal collection in all studies, mice were deeply anesthetized with isoflurane. Prior to perfusion, whole blood was collected from the right ventricle *via* cardiac puncture (23 g needle) into lithium heparin-containing vials (BD microtainer, Becton Dickinson 365958) for plasma collection. In some cases, whole blood was also collected into EDTA-containing vials (Minicollect tube, Greiner Bio-One 450480) for peripheral blood mononuclear cell isolation. Blood and plasma tubes were subjected to centrifugation within 60 min of collection and were stored on ice in the interim. For perfusion, the right atrium was opened, and mice were transcardially perfused with ice cold phosphate buffered saline (PBS) through a 25 g needle. Brains were collected, flash-frozen intact on dry ice, and stored at −80°C for later frozen dissection. Complementary hippocampal-containing coronal sections from the left and right hemispheres were used for qPCR and Luminex assays, respectively, while a coronal section of the frontal cortex from the left hemisphere was used for western blot. Whole blood was centrifuged (BD microtainer, 3,000 g for 10 min) for plasma separation and was frozen on dry ice and stored at −80°C.

### Quantitative RT-PCR

Total RNA was isolated from brain tissue sections containing the hippocampus using the RNeasy Lipid Tissue Mini Kit (Qiagen, Germantown, MD). One microgram of total RNA was transcribed into cDNA (Superscript III, Invitrogen, Waltham, MA). PCR was performed in triplicate using TaqMan gene expression Master Mix (Applied Biosystems, Waltham, MA) and validated TaqMan gene expression assays for *Tnfα* (Mm00443258_m1), *Il1β* (Mm00434228_m1), *Il6* (Mm00446190_m1), *Lamp1* (Mm00495262_m1), *CD68* (Mm03047340_m1), and glyceraldehyde-3-phosphate dehydrogenase (*Gapdh*; Mm99999915_g1). Amplification was performed using a StepOnePlus system (Applied Biosystems, Waltham, MA). Fold changes of expression relative to control were determined after normalization to *Gapdh*. Relative quantification and fold change were calculated by the comparative CT method (Schmittgen and Livak, 2008).

### Western blot

Tissue from the left frontal cortex was homogenized in 2–5 mL of S buffer (0.32 M sucrose, 1 mM EDTA, and 5 mM Tris, pH 7.4) using a Potter-ELV tissue grinder at 800 rpm (8 strokes). The homogenate was spun at 1,000 g for 10 min at 4°C. The supernatant was collected and spun at 10,000 g for 20 min at 4°C. The pellet was resuspended in T-PER (Tissue Protein Extraction Reagent; Life Technologies, Cat: 78510 Carlsbad, CA) with Protease Inhibitor (Life Technologies, Cat: 78430, Carlsbad, CA) and Phosphatase Inhibitor Cocktails (Abcam, Cat: ab201113, ab201112, ab201114, Cambridge, United Kingdom). Samples were homogenized on ice by sonication using an ultrasonic probe homogenizer (Omni International, Kennesaw, GA) and centrifuged at 12,000 g for 10 min at 4°C to obtain the soluble fraction containing the plasma membrane. The protein concentration was determined using the BCA protein assay kit (Pierce, Cat: 23227 Rockford, IL). Samples were boiled, loaded (20 μg/well), and resolved by gel electrophoresis under denaturing conditions using 10% Bis-Tris, 1.0 mm, Mini Protein Gel, 17-well (Life Technologies, Cat: NW00107BOX Carlsbad, CA). The protein was transferred to a polyvinylidene difluoride membrane (Abcam, Cat: ab133411, Cambridge, United Kingdom) and incubated in Intercept® (TBS) Blocking Buffer (Li-cor, Cat: 927–60,001, Lincoln, NE) for 1 h at room temperature. The membranes were incubated at 4°C overnight with AKT (1:2,000), phospho AKT (1:1,000), GSK-3β (1:1,000), phospho-GSK-3β (1:1,000), ERK1/2 (1:1,000), phospho ERK1/2 (1:2,000), alpha-tubulin (1:10,000), ATG5 (1:1,000), phospho p70S6k (1:1,000), and p70S6k (1:1,000) primary antibodies. The following day, membranes were washed (3 × 5 min) with 0.01% Tween-20 in 1x TBS and incubated for 1 h at room temperature with IRDye® IgG Secondary Antibody (goat anti-mouse Cat: 926-68070, goat anti-rabbit Cat: 926-32211; 1:10,000, Li-cor, Lincoln, NE). Following secondary antibody incubation, membranes were washed (3 × 5 min) with 0.01% Tween-20 in 1x TBS. Membranes were then scanned with the Sapphire Biomolecular Imager (Azure Biosystems, Dublin, CA) in the appropriate wavelengths. AzureSpot software (Azure Biosystems) was used for densitometry analysis of target protein levels and normalized to the internal level of tubulin for each sample as control.

### Multiplex mouse cytokine assay

Multiplex tissue cytokines were analyzed in plasma and brain homogenate from hippocampal containing sections using a Luminex 48-plex (ProcartaPlex, Invitrogen, Carlsbad, CA, United States) mouse cytokine assay. The Luminex assay was performed at the Human Immune Monitoring Center at Stanford University following manufacturer instructions. Briefly, hippocampal tissue was homogenized in RIPA buffer containing proteinase inhibitor (1:100) by pulling tissue through a 23 g needle (15×) and then sonicating the tissue using 3 × 3 second pulses. Homogenate was spun at 14,000 g for 10 min, and protein concentrations were determined by Pierce BCA assay. Brain homogenates were diluted to a common concentration of 6 μg/uL. Plasma samples were diluted 1:3. Plasma and brain homogenate samples were run in a singlet on a 96 well plate alongside standard curve and quality control calibration samples.

### Statistics

Statistical analyses were performed with GraphPad Prism 9.0. The ROUT method was used to determine statistical outliers, which were subsequently excluded from analyses. One-way ANOVA followed by Sidak multiple comparisons test was used to compare select treatment groups. Differences between the groups were considered statistically significant at *p* < 0.05, but results with effects approaching this threshold are also discussed as relevant trends (Amrhein et al., 2019; Wasserstein et al., 2019).

## Data availability statement

The raw data supporting the conclusions of this article will be made available by the authors, without undue reservation.

## Ethics statement

The animal study was reviewed and approved by Stanford University Administrative Panel for Laboratory Animal Care.

## Author contributions

AB and MS designed, supervised, and funded the study and helped to write and edit the manuscript. SS, PC, GO, and LV performed animal experiments and/or *ex vivo* assays. SS and ED performed data analysis. SS, ED, and JF helped to write and edit the manuscript. JL contributed to revisions of the manuscript. All authors contributed to the article and approved the submitted version.

## Funding

We thank the NIH for funding this work with a Director’s Pioneer Award to AB, grant # 1DP1 OD029517. AB also acknowledges funding from Stanford University’s Discovery Innovation Fund, the Cisco University Research Program Fund, and the Silicon Valley Community Foundation, Stephen Pearse, and James J. Truchard and the Truchard Foundation. Additionally, we thank Robert Mullen, who provided a generous gift to help fund the first phase of this research study. Finally, portions of the study were conducted by the Stanford Behavioral and Functional Neuroscience Laboratory, which is supported by an NIH S10 Shared Instrumentation for Animal Research under award number 1S10OD030452-01.

## Conflict of interest

AB and JF are co-founders and members of the board of directors of Reversal Solutions, Inc. and hold shares of stock in the company.

The remaining authors declare that the research was conducted in the absence of any commercial or financial relationships that could be construed as a potential conflict of interest.

## Publisher’s note

All claims expressed in this article are solely those of the authors and do not necessarily represent those of their affiliated organizations, or those of the publisher, the editors and the reviewers. Any product that may be evaluated in this article, or claim that may be made by its manufacturer, is not guaranteed or endorsed by the publisher.

## Abbreviations

PBM: Photobiomodulation
LPS: Lipopolysaccharide
NIR: Near-infrared
RL: Red light/NIR
RLG: Red light/NIR with 40 Hz gamma flicker
NL: No light
IP: Intraperitoneal
