# Supplementary material for: The anti-inflammatory effects of photobiomodulation are mediated by cytokines: Evidence from a mouse model of inflammation
Source: Front Neurosci. 2023 Apr 6;17:1150156. doi: 10.3389/fnins.2023.1150156 (PMC10115964; doi:10.3389/fnins.2023.1150156)
Supplement: Supplementary file 1 [file Data_Sheet_1.docx]

Supplementary Material

The anti-inflammatory effects of photobiomodulation are mediated by cytokines: evidence from a mouse model of inflammation

**Shirin Shamloo^1,2,†^, Erwin Defensor^1,2,†^, Peter Ciari^2^, Gaku Ogawa^2^, Laura Vidano^2^, Jennifer S. Lin^1,2^, John A. Fortkort^1^, Mehrdad Shamloo^2,*^, Annelise E. Barron^1,*^**

^1^Stanford University, Schools of Medicine and of Engineering, Department of Bioengineering, Shriram Center, Stanford, CA, USA

^2^Stanford University, School of Medicine, Department of Neurosurgery, Stanford, CA, USA

^†^These authors contributed equally to this work and share first authorship

**^*^Co-corresponding authors:** Annelise E. Barron, [aebarron@stanford.edu](mailto:aebarron@stanford.edu) ; Mehrdad Shamloo, [shamloo@stanford.edu](mailto:shamloo@stanford.edu)

**Supplementary Materials and Methods**

*PBM Technical Specifications*

The Auragen LEDs used in this study are from SunLED Company, LLC, Walnut, CA. The optical and performance properties of the LEDs are summarized in **Tables S1** and **S2**, respectively, which are derived from the manufacturer’s data sheets for the NIR LED (Part number: XTHI30W), red LED (Part number: XLM2ACR11W), and blue LED (XLFBB11W). Unless otherwise noted, all parameters were measured at 25°C. Graphs of the radiant intensities of and spatial distributions for the NIR, red, and blue LEDs are available from the manufacturer’s detailed data sheets. Each LED is equipped with a clear lens.

**Table S1: Auragen LED Optical Properties**

| Diode | Wavelength of Peak  Emission (Typ.)  (IF=20mA)  (nm) | Wavelength of Dominant  Emission (Typ.)  (IF=20mA)  (nm) | Spectral Line Full Width  At Half-Maximum  (IF=20mA)  (nm) | Emitting  Material | Radiant Intensity  (Po=mW/sr)  @20mA | Luminous Intensity  (IF=20mA)/  Wavelength (nm)^[[1]](#footnote-1)^ | Viewing  Angle  2θ 1/2 |
| --- | --- | --- | --- | --- | --- | --- | --- |
| NIR | 880 | - | 50 | GaAlAs | 15 | - | 50° |
| Red | 640 | 625 | 25 | AlGaInP | - | 4090 | 30° |
| Blue | 465 | 470 | 22 | InGaN | - | 3690/465 | 30° |

**Table S2: Auragen LED Performance Properties**

| Diode | Reverse Voltage  (V) | Forward Voltage (Typ.)  (IF=20mA)  (V) | Forward Voltage (Max.)  (IF=20mA)  (V) | Forward Current (mA) | Reverse Current (Max.)  (VR=5V)  (µA) | Forward Current (Peak)  1/10 Duty Cycle  0.1ms Pulse Width (mA) | Power  Dissipation  (mW) |
| --- | --- | --- | --- | --- | --- | --- | --- |
| NIR | 5 | 1.3 | 1.6 | 50 | 10 | 1200 | 85 |
| Red | 5 | 2.2 | 2.8 | 30 | 10 | 150 | 84 |
| Blue | 5 | 3.3 | 4 | 30 | 50 | 100 | 120 |

In terms of radiant intensity and spatial distribution, the LEDs in the Auragen unit provide a narrowly focused beam. In the case of the NIR LEDs, for example, this beam delivers > 99% of the radiant intensity within a five degree from the center cone and > 50% of the radiant energy at 22° off center (refer to figure on “NIR Radiant Intensity and Spatial Distribution” at https://reversalsolutions.com/a/blog/science-of-light-therapy). As seen in **Table S1**, the NIR LEDs are designed to provide a radiant intensity of 15 mW/sr.

# Supplementary Discussion

AKT, ERK, GSK3β, p70S6K, and ATG5 are involved in the regulation of several biological processes, including cell growth, survival, proliferation, and/or autophagy. LPS did not induce phosphorylation of AKT, ERK, GSK3β, p70S6K, or ATG5 when measured by western blot in mouse forebrain tissue. Thus, the attenuating effects of PBM on the activation of the associated pathways could not be determined in this study. Furthermore, red light (red/NIR+Veh) or red light with 40 Hz flicker (red/NIR Gamma+Veh) did not significantly alter AKT, ERK, GSK3β, p70S6K, or ATG5 as indicated by comparison of these conditions to No Light+Veh.

# Supplementary Figures

**Fig. S1.** Custom-built isolation chambers prevent light and light flicker contamination between chambers.

**Fig. S2.** **Luminex analysis of select cytokines in the plasma.** The RL+Veh and RLG+Veh groups both showed decreased EOTAXIN, while the RL+Veh group also showed increased MCP-3, and the RLG+Veh group showed increased IL-17A and IL-23, when compared to the NL+Veh group. Consistent with the expected induction of inflammation by LPS, several cytokines were increased in the NL+LPS group when compared to the NL+Veh group. The RL+LPS and RLG+LPS groups both showed increased IL-10, as well as decreased IL-1β, IL-22, and IL-7Rα when compared to NL+LPS. The RL+LPS group also showed decreased IL-18 and increased MIP-1β, while the RLG+LPS group showed decreased IP-10, IFN-γ, RANTES, MCP-1, IL-2Rα, and BTC, when compared to the NL+LPS group. Data are presented as mean ± SEM. *p<0.05, **p<0.01, ***p<0.001, ****p<0.0001.

**Fig. S3.** **Luminex analysis of select cytokines in hippocampus-containing brain tissue.** The RLG+Veh group showed a reduction in IL-7Rα and IL-22 when compared to the NL+Veh group. LPS induction was evident in increased ST2, IFN-α, sRANKL, MCP-1, and IL-15 in the NL+LPS group, when compared to the NL+Veh group. RL+LPS and RLG+LPS groups both showed a reduction in IL-33, IFN-α, IL-7Rα, and IL-27, while the RLG+LPS group also showed a reduction of sRANKL, MCP-1, and IL-15, when compared to the NL+LPS group. Data are presented as mean ± SEM. *p<.05, **p<0.01, ****p<0.0001.

**Fig. S4.** Western blot analysis of the left forebrain shows no significant differences between treatment groups in levels of phospho-AKT or phospho-GSK3β. Scatter bar graphs depict the quantification of a western blot for pAKT relative to AKT as well as pGSK3β relative to GSK3β.

**Fig. S5.** Western blot analysis of the left forebrain shows no significant differences between treatment groups in levels of phospho-ERK 1/2. The scatter bar graph depicts the quantification of the western blot for pERK 1/2 relative to ERK 1/2.

**Fig. S6.** Western blot analysis of the left forebrain shows no significant differences between treatment groups in levels of ATG5. The scatter bar graph depicts the quantification of the western blot for ATG5 relative to tubulin.

**Fig. S7.** Western blot analysis of the left forebrain shows no significant differences between treatment groups in levels of phospho-p70S6k. Scatter bar graph depicts quantification of the western blot for phospho-p70S6k relative to p70S6k.

1. Luminous intensity value and wavelength are in accordance with CIE127-2007 standards. [↑](#footnote-ref-1)
