# Supplementary figures and images for: The anti-inflammatory effects of photobiomodulation are mediated by cytokines: Evidence from a mouse model of inflammation
Source: Front Neurosci. 2023 Apr 6;17:1150156. doi: 10.3389/fnins.2023.1150156 (PMC10115964; doi:10.3389/fnins.2023.1150156)

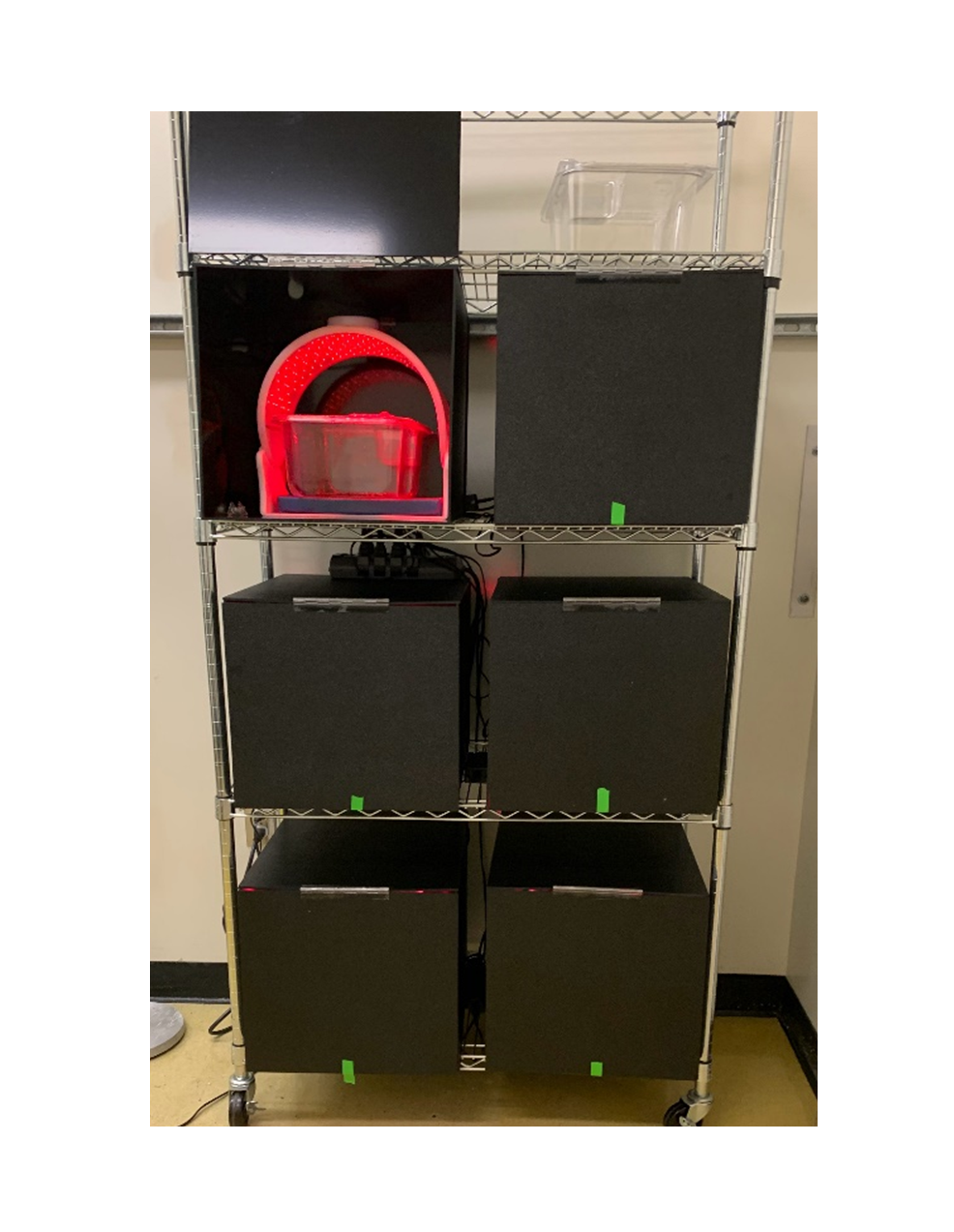

Supplement: Supplementary file 2 [file Image_1.tif]

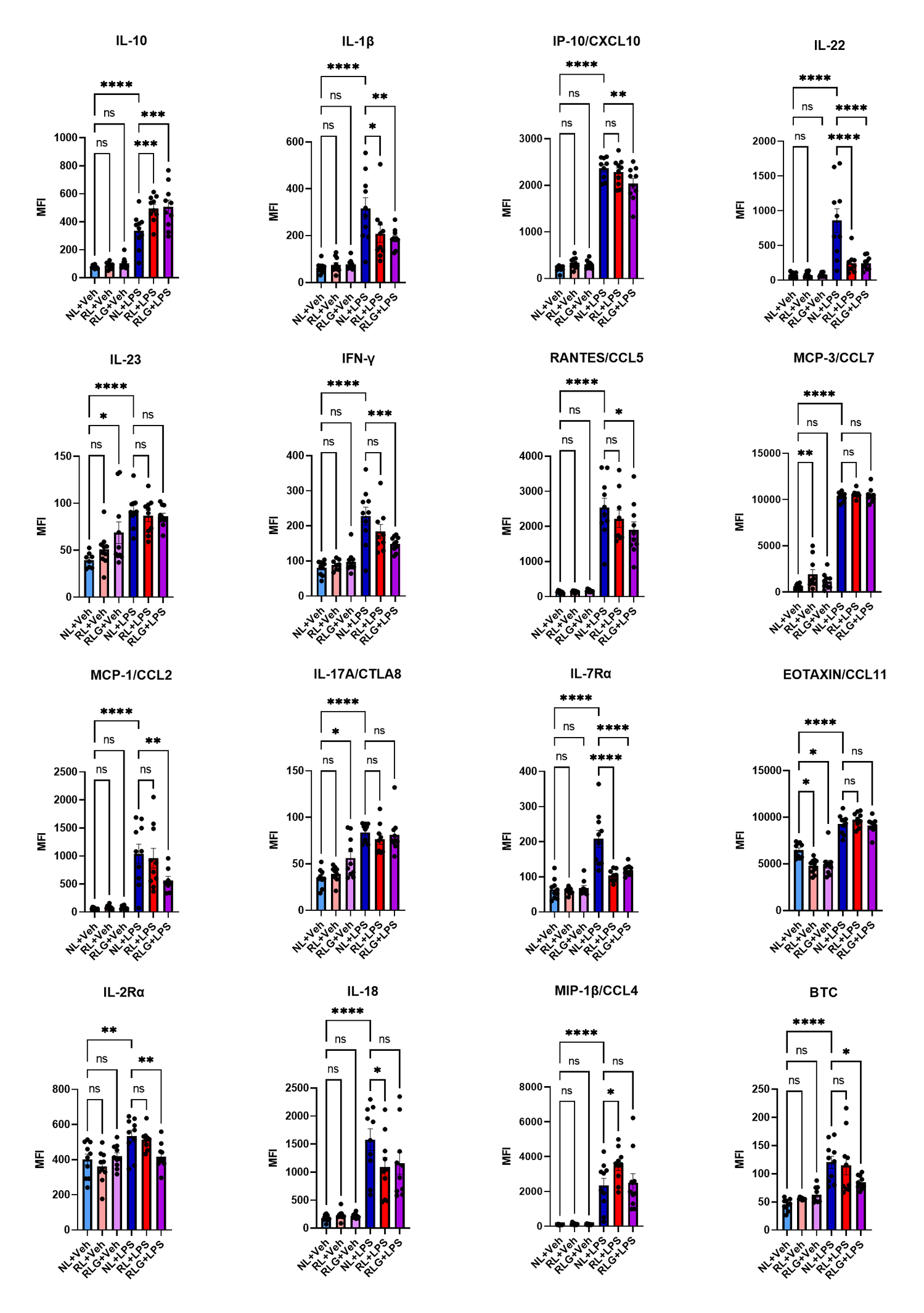

Supplement: Supplementary file 3 [file Image_2.tif]

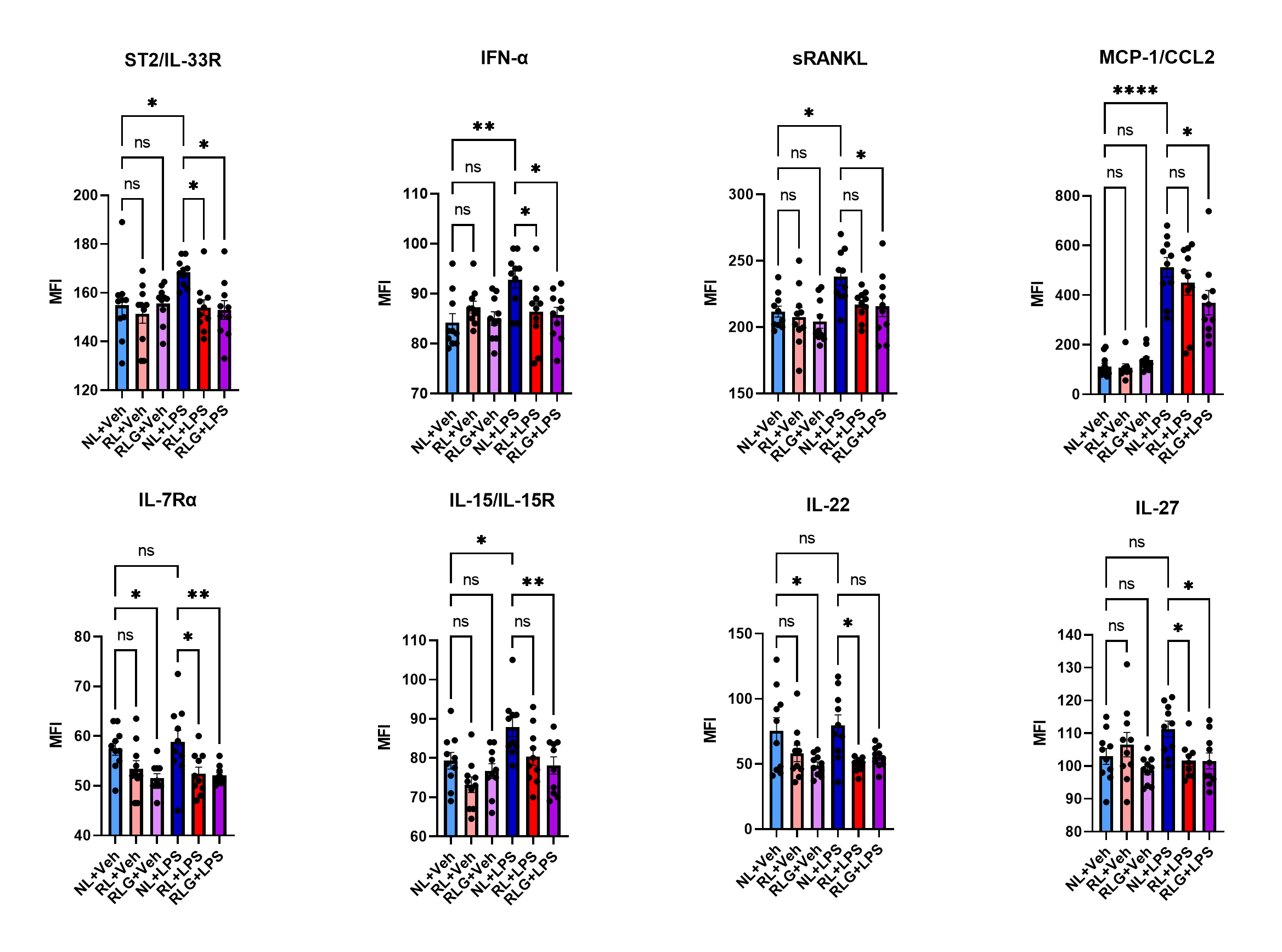

Supplement: Supplementary file 4 [file Image_3.tif]

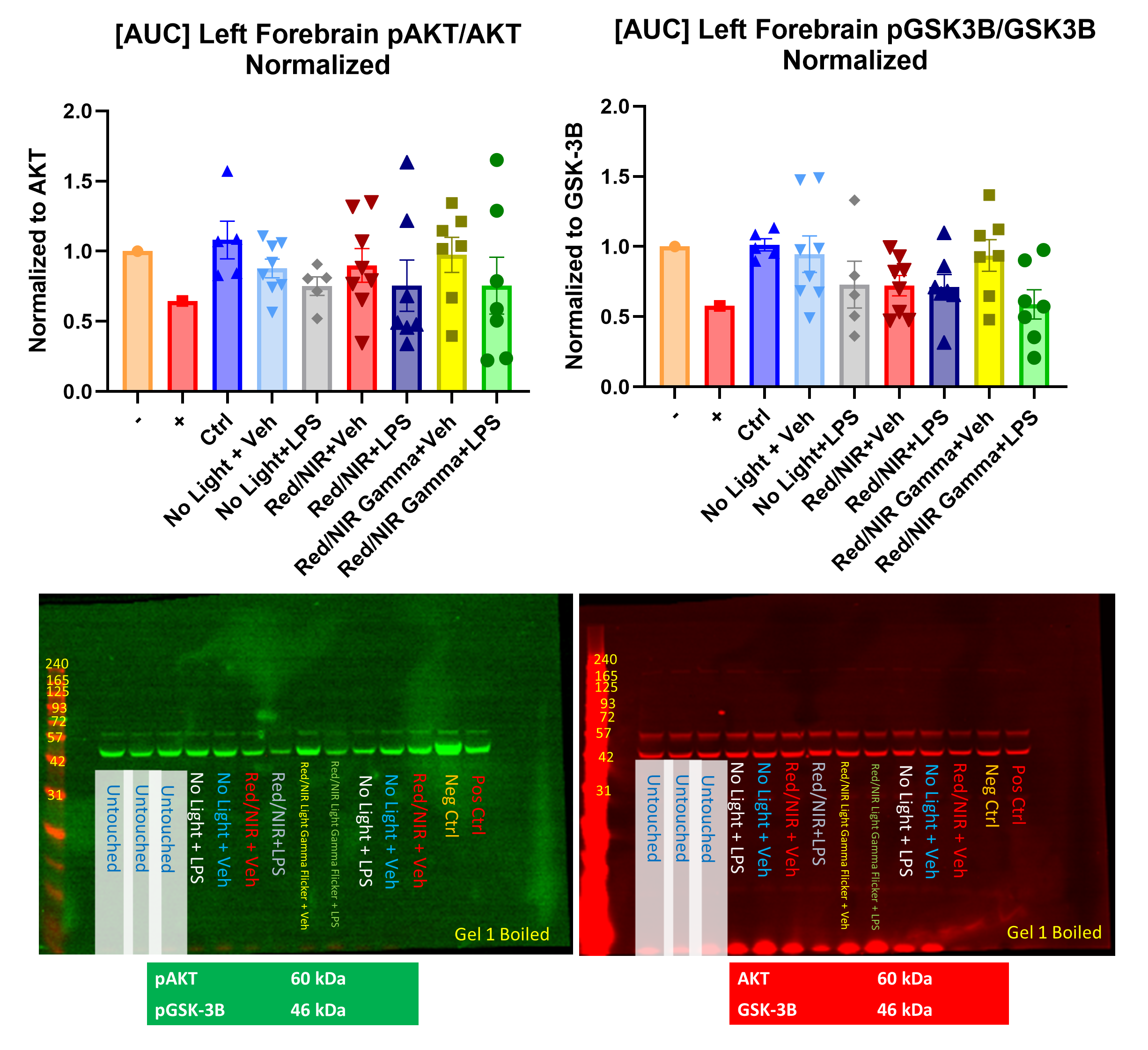

Supplement: Supplementary file 5 [file Image_4.tif]

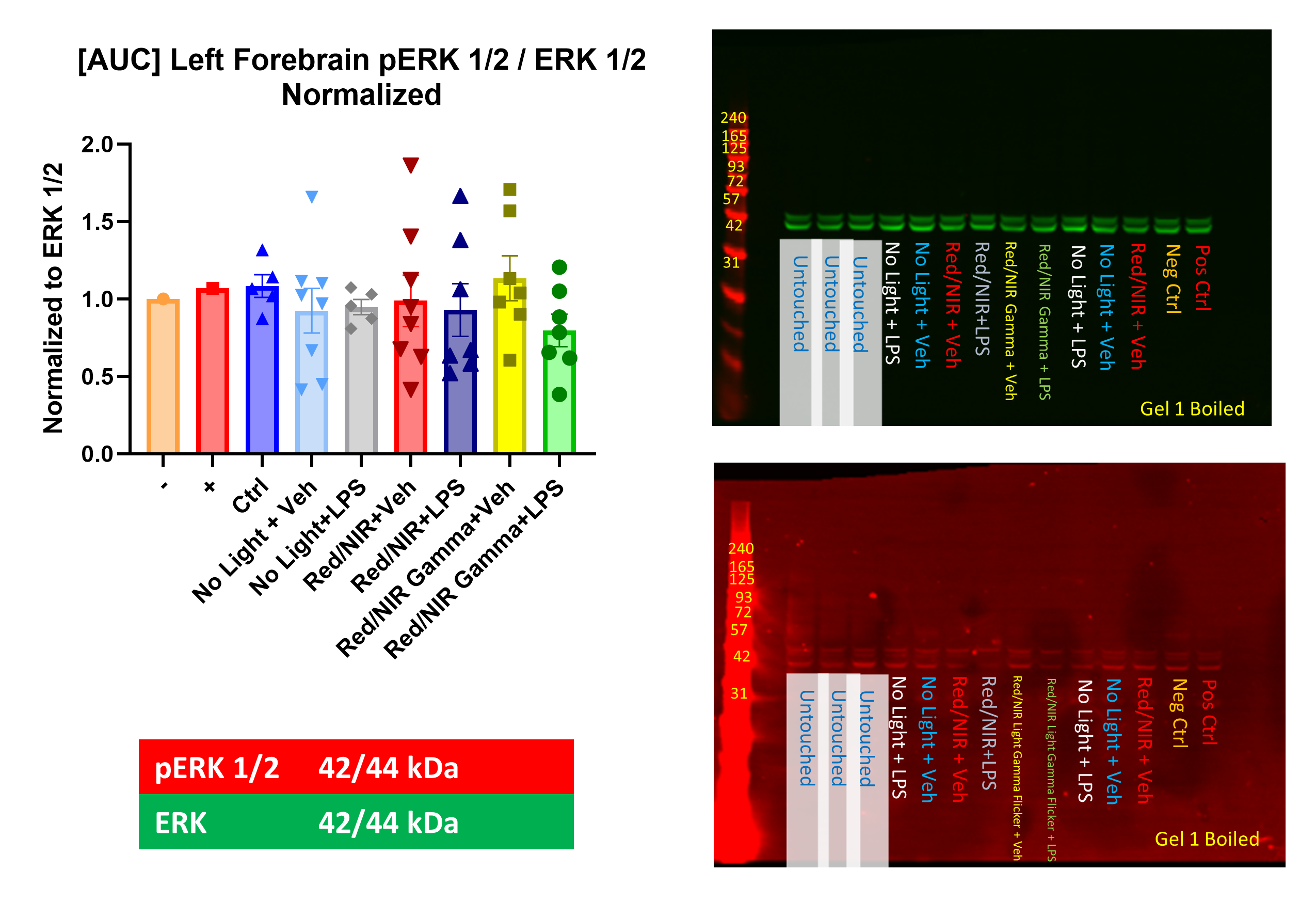

Supplement: Supplementary file 6 [file Image_5.tif]

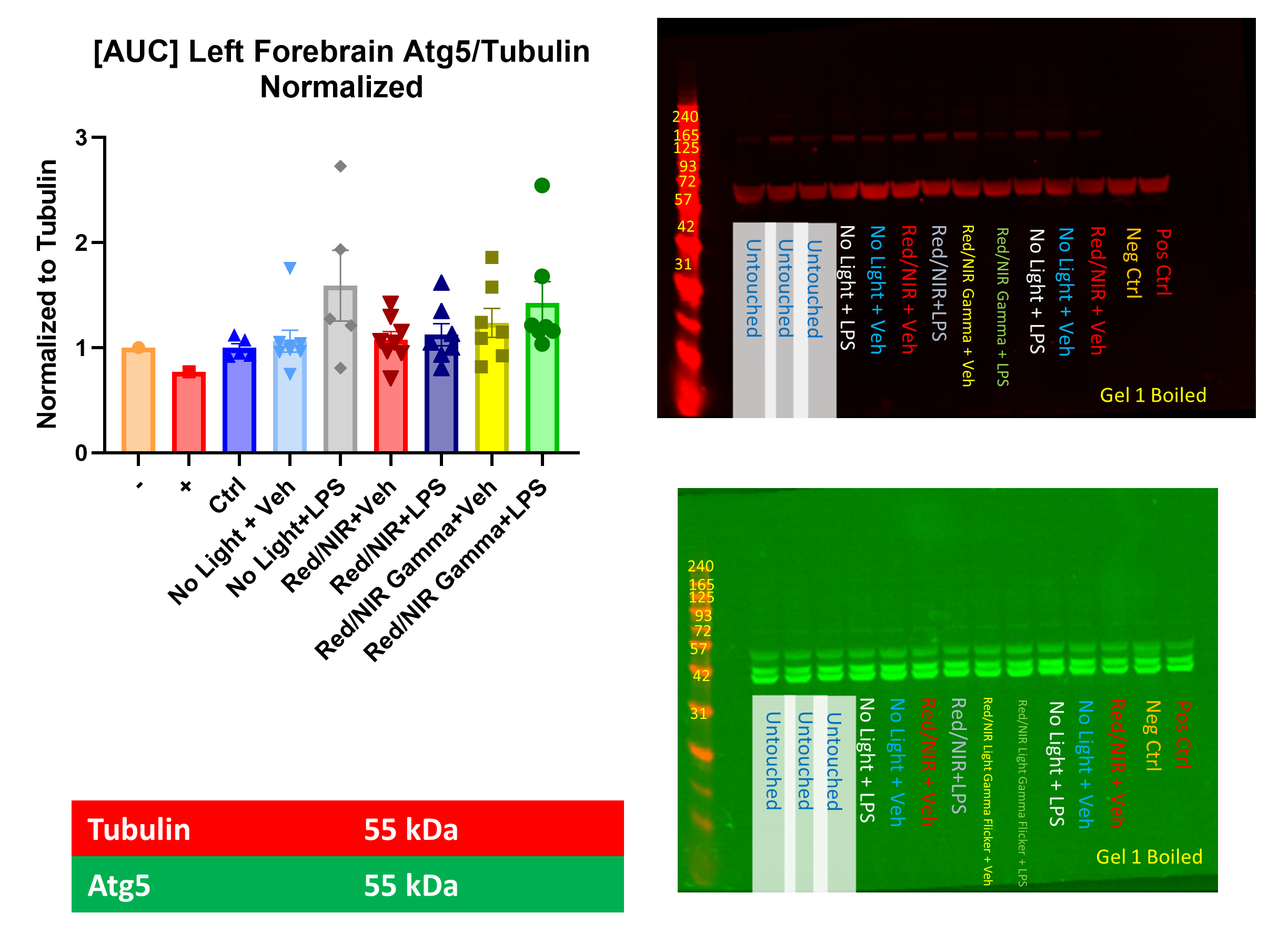

Supplement: Supplementary file 7 [file Image_6.tif]

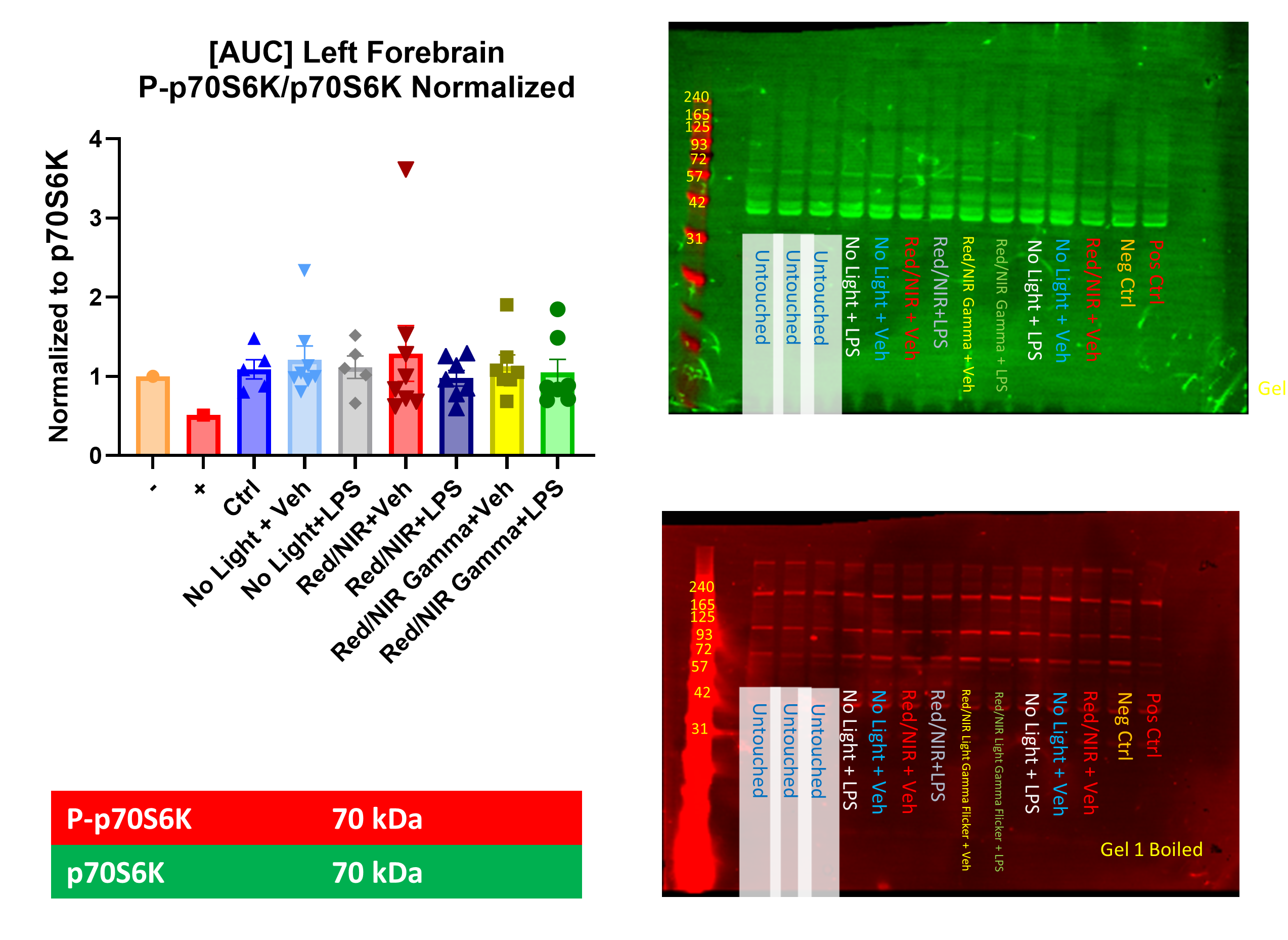

Supplement: Supplementary file 8 [file Image_7.tif]
